# Self-Reported Acceptance of a Wearable Activity Monitor in Persons With Stroke: Usability Study

**DOI:** 10.2196/70007

**Published:** 2025-12-23

**Authors:** Jamie Nam, Grace C Bellinger, Junyao Li, Margaret A French, Ryan T Roemmich

**Affiliations:** 1 Department of Physical Medicine and Rehabilitation, Johns Hopkins University School of Medicine, Baltimore, MD, United States; 2 Feinberg School of Medicine, Northwestern University, Chicago, IL, United States; 3 Department of Physical Therapy and Athletic Training, University of Utah, Salt Lake City, UT, United States; 4 Center for Movement Studies, Kennedy Krieger Institute, Room 240, 716 N Broadway, Baltimore, MD, 21205, United States, 1 4439232717

**Keywords:** stroke, wearables, mobility, technology acceptance, physical activity, remote monitoring, rehabilitation

## Abstract

**Background:**

Wearable activity monitors offer clinicians and researchers accessible, scalable, and cost-effective tools for continuous remote monitoring of functional status. These technologies can complement traditional clinical outcome measures by providing detailed, minute-by-minute, remotely collected data on a wide array of biometrics, including physical activity and heart rate. There is significant potential for the use of these devices in rehabilitation after stroke if individuals will wear and use the devices; however, the acceptance of these devices by persons with stroke is not well understood.

**Objective:**

This study investigated the self-reported acceptance of a commercially available, wrist-worn wearable activity monitor (the Fitbit Inspire 2; Fitbit Inc) for remote monitoring of physical activity and heart rate in persons with stroke. We also assessed relationships between reported acceptance and adherence to wearing the device.

**Methods:**

Sixty-five participants with stroke wore a Fitbit Inspire 2 for 3 months, at which point we assessed acceptance using the Technology Acceptance Questionnaire (TAQ), which includes 7 dimensions: perceived usefulness, perceived ease of use, equipment characteristics, privacy concerns, perceived risks, facilitating conditions, and subjective norm. We then performed Spearman correlations to assess relationships between acceptance and adherence to device wear, calculated as both the percentage of daily wear time and the percentage of valid days the device was worn during the 3 weeks preceding TAQ administration.

**Results:**

Most participants reported generally agreeable responses, with high overall total TAQ scores across all 7 dimensions, indicating strong acceptance of the device; “Agree” was the median response to 29 of the 31 TAQ statements. Participants generally found the device beneficial for their health, efficient for monitoring, easy to use and to don and doff, and unintrusive to daily life. However, participant responses on the TAQ did not show significant positive correlations with measures of actual device wear time (all *P*>.05).

**Conclusions:**

This study demonstrates generally high self-reported acceptance of the Fitbit Inspire 2 among persons with stroke. Participants reported general agreement across all 7 TAQ dimensions, with minimal concerns interpreted as being directly relatable to poststroke motor impairment (eg, donning and doffing the device, using it independently). However, the high self-reported acceptance scores did not correlate positively with measures of real-world device wear. Accordingly, it should not be assumed that persons with stroke will adhere to wearing these devices simply because they report high acceptability.

## Introduction

### Background

Wearable devices have the potential to advance how clinicians and researchers measure functional status by offering accessible, scalable, and cost-effective remote monitoring tools [1,2]. Traditional outcome measures provide only a discrete snapshot of an individual’s functional status [3-5]; emerging wearable technologies have the potential to address this issue by providing minute-by-minute, longitudinal data on physical activity, heart rate, and many other biometrics from daily life that extend beyond clinical or laboratory settings. The use of wearable devices for remote monitoring could offer additional observational data on functional status measured directly in an individual’s daily environment.

Notably, wearable devices could provide valuable insight into recovery following neurologic damage (eg, after stroke) by enabling granular, longitudinal assessment of activity, one of the components of the World Health Organization’s International Classification of Functioning, Disability, and Health model [6]. Approximately 80% of persons with stroke experience some form of motor impairment, and around 50% continue to have significant functional limitations 6 months poststroke [7-9]. These limitations often translate into reduced daily activity, as persons with stroke generally walk approximately half as many steps each day as individuals without stroke [10]. Wearable devices provide an opportunity to monitor these functional changes remotely and to generate insights on daily physical activity. However, the ability to perform remote monitoring after stroke using wearable devices is dependent on how well persons with stroke accept these devices.

### Previous Work

Wearable activity monitors such as Fitbit devices generally show high acceptance among healthy individuals [11] and across a range of patient groups, including older adults with cognitive and motor impairment [12,13]. For older adults with cognitive impairment, Fitbit-based interventions may improve motivation for physical activity and sleep, but success is dependent on interfaces that are easy to use [14], reduce cognitive load [15], and are beneficial to the user [14]. In studies of persons with minimal motor impairment, the lack of sustained Fitbit usage and challenges to acceptance have largely been attributed to technical issues—including empty batteries, broken devices, or lost devices [16]—rather than usability concerns. For example, users with multiple sclerosis reported frustration when syncing data between devices [17].

To measure acceptance of wearable technologies across diverse clinical populations, researchers have extensively used the technology acceptance model due to its simplicity and strong empirical support [18,19], with the Technology Acceptance Questionnaire (TAQ) [13] serving as an extension of this framework with additional factors related to user acceptance. Numerous studies have validated this framework across many contexts, demonstrating its predictive power in understanding technology adoption behaviors [18-22]. While wearable activity monitors have demonstrated varying levels of acceptance across the general population, their perceived usability and effectiveness in individuals with stroke remain less explored, which prompted our use of the TAQ to assess their suitability in this specific patient group [23].

### Objective

We aimed to assess the acceptance of wearable devices in persons with stroke. We also examined the relationship between acceptance and adherence to wearing the Fitbit device. We studied Fitbit devices specifically because they have been used extensively for remote monitoring of step count, heart rate, and energy expenditure—among other metrics—in many populations [24]. We hypothesized that (1) acceptance of the Fitbit devices would be variable across persons with stroke but would generally indicate that these technologies are acceptable, useful, and easy to use, and (2) acceptance would be significantly associated with real-world adherence to wearing the Fitbit device.

## Methods

### Recruitment

We recruited persons with stroke from the outpatient rehabilitation clinics at Johns Hopkins Hospital through clinician referrals and MyChart (Epic Systems) messages. The inclusion criteria for this study were: age 18 years and older; history of stroke (confirmed by International Classification of Diseases, Tenth Revision [ICD-10] codes); ownership of a smartphone and in-home Wi-Fi access; walking as a primary form of mobility (with assistive devices allowed); and ability and willingness to install the Fitbit mobile app on a smartphone.

After obtaining consent, the study team asked participants to report their age, sex, race, ethnicity, handedness, and degree of impairment. We then mailed a Fitbit Inspire 2 (Fitbit Inc) to participants and asked them to wear it for 1 year as part of a larger study [25]. This study focuses on a subanalysis of the first 3 months of device use, during which the TAQ was administered to assess participants’ experiences and perceptions of the device at the 3-month time point. As an incentive, we allowed participants to keep the Fitbit following completion of the study.

### Study Instructions

We instructed participants to wear the Fitbit on their nonparetic (ie, less impaired) wrist throughout the entire day, including during sleep; if participants had difficulty donning the device and lacked available assistance, we permitted them to wear it on the paretic wrist. We documented the paretic side and the wrist on which the Fitbit was worn. Then, we guided the participants over the phone to set up the device and install the Fitbit app on their smartphone. We instructed participants to remove the device only when showering or charging it. We also asked them to synchronize the device at least once per day using the Fitbit smartphone app. Data from the Fitbit were extracted by a custom-built app, described elsewhere [25,26]. The study team incorporated notifications and reminders to assist with adherence to wearing the Fitbit, as detailed in our previous work [25].

After a participant was enrolled for 3 months, the study team attempted to contact the participant up to 3 times to administer the TAQ, our metric of acceptance. Each contact attempt was documented in Research Electronic Data Capture (REDCap; Vanderbilt University), which included the date and time of the call, the outcome of the attempt (eg, reached, voicemail, and no answer), the participant’s response to the assessment, and any notes or follow-up actions required. If the participant was reached, the team member administered the questionnaire verbally. However, if the participant was not reached after 3 attempts, no further attempts were made to administer the TAQ.

### Technology Acceptance Questionnaire

We used the TAQ—which includes dimensions for perceived usefulness (PU), perceived ease of use (PEOU), equipment characteristics, privacy concerns, perceived risks, facilitating conditions, and subjective norm—as established by Puri et al [13]. The full questionnaire is described in Puri et al [13]. The TAQ consisted of 31 statements rated on a 5-point Likert scale where participants indicated their levels of agreement or disagreement with each statement. These 31 statements covered dimensions of PU (n=5 statements), PEOU (n=7 statements), facilitating conditions (n=2 statements), subjective norm (n=3 statements), equipment characteristics (n=8 statements), privacy concerns (n=3 statements), and perceived risks (n=3 statements). We summed the scores within each dimension (“Strongly disagree”=1 point, “Disagree”=2 points, “Neutral”=3 points, “Agree”=4 points, “Strongly agree”=5 points). We note that, to ensure the reliability and validity of the measure, certain statements in the TAQ were negatively framed to mitigate acquiescence bias [27]. As is standard, we minimized possible response biases in which higher numerical values represent lower agreement by applying a reverse-coding procedure to these statements: each affected score was standardized by subtracting it from 6 for statements 2, 10, 17, 25, and 27. This was done before summing the scores within each dimension. We then calculated the total TAQ score by summing the scores from all items across all dimensions. In addition to the 31 statements, there are also 6 multiple-choice questions addressing various aspects of device use that are not assigned to any dimension. Finally, we also provided participants with the opportunity to share open-ended comments about their experiences using the devices.

### Fitbit Adherence

To assess adherence, we analyzed Fitbit data from the 21 days immediately preceding completion of the TAQ. This timeframe was selected because the TAQ items specifically reference user perceptions of their Fitbit over the previous 3 weeks. We identified Fitbit wear minutes using the accelerometry package [28] in R (R Foundation for Statistical Computing) [29]. We calculated 2 primary adherence metrics. First, we calculated the average percentage of time the device was worn each day by dividing the number of wear minutes by the total minutes in a day (1440), multiplying by 100, and averaging the daily percentages across the 21-day period. Second, we calculated the percentage of valid wear days as the number of days within the 3-week window with at least 75% of 24-hour wear time (ie, 1080 or more minutes). The number of valid days was then divided by 21 (the total days in the assessment window) and multiplied by 100 to determine the percentage of valid days.

### Statistical Analysis

We calculated descriptive statistics for the 37 individual items of the TAQ (inclusive of the 31 Likert scale statements and 6 additional multiple-choice questions), the summed scores on each of the 7 dimensions from the TAQ, and the summed total score of the TAQ. We also report descriptive statistics for the metrics of adherence. To assess relationships between Fitbit adherence and TAQ responses, we used Spearman correlations (due to the ordinal nature of the TAQ responses). We performed all statistical analyses using R (version 4.4.1; R Foundation for Statistical Computing) [29] with *α*=.05.

### Ethical Considerations

All participants provided oral consent, as approved by the Institutional Review Board at the Johns Hopkins University School of Medicine (IRB00247292). All data were deidentified. We did not provide participants with monetary compensation; however, they were allowed to keep their Fitbit devices following completion of the study as compensation for their participation.

## Results

### Participants

**Figure 1.**
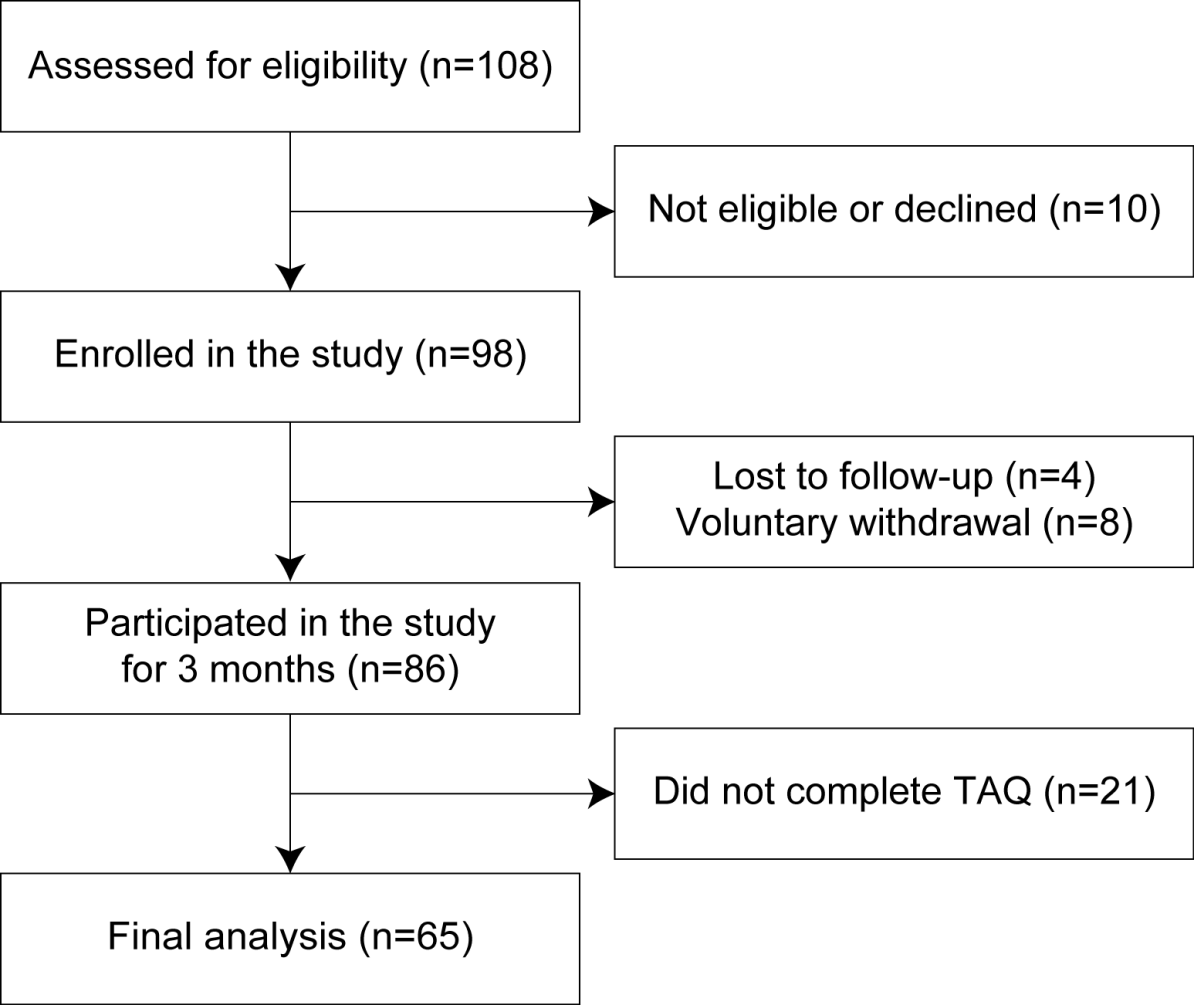
Flow diagram showing participant enrollment and participation at various stages of the study. TAQ: Technology Acceptance Questionnaire.

Of the 108 persons with stroke contacted, 98 participants enrolled in this study (8 individuals either did not meet the inclusion criteria or declined to participate and 2 individuals enrolled in the larger study but declined the Fitbit portion). Among the enrolled participants, 4 were lost to follow-up (ie, they could not be reached despite repeated attempts during the 3-month study period) and 8 withdrew voluntarily (ie, they chose to discontinue their involvement after enrolling). Furthermore, 21 participants did not complete the TAQ 3 months postenrollment. As a result, we included 65 participants with stroke in the final analysis. We present a participant enrollment flow diagram in Figure 1 and summarize the characteristics of participants included and excluded in the analysis in Table 1.

**Table 1.** Study participant characteristics.

Characteristic	Included participants (n=65)	Excluded participants (n=35)^a^
Age (years), mean (SD)	62.4 (12.4)	60.5 (12.5)
Days poststroke at date of enrollment, mean (SD)	1053.9 (1664.1)	230.1 (367.0)
Days between the date of enrollment and the date of TAQ completion, mean (SD)	127.8 (42.9)	—^b^
Sex, n (%)		
* *Male	37 (56.9)	20 (57.1)
* *Female	28 (43.1)	15 (42.9)
Race, n (%)		
American Indian or Alaska Native	2 (3.1)	0 (0.0)
Asian	3 (4.6)	1 (2.9)
Black or African American	21 (32.3)	22 (62.9)
White or Caucasian	38 (58.5)	10 (28.6)
Multiple	1 (1.5)	2 (5.7)
Ethnicity, n (%)		
Hispanic or Latino	4 (6.2)	1 (2.9)
Not Hispanic or Latino	61 (93.8)	34 (97.1)
Use of an assistive device for walking, n (%)		
Yes	28 (43.1)	13 (37.1)
No	37 (56.9)	21 (60.0)
No response	0 (0.0)	1 (2.9)
Able to move the paretic arm, n (%)		
Yes	57 (87.7)	30 (85.7)
No	8 (12.3)	3 (8.6)
No response	0 (0.0)	2 (5.7)
Able to bring a hand from the lap to the table, then to top of chest, and reach for object above the table surface, n (%)		
Yes	56 (86.2)	31 (88.6)
No	8 (12.3)	2 (5.7)
Unsure	1 (1.5)	0 (0.0)
No response	0 (0.0)	2 (5.7)
Severe shoulder pain that limits the ability to move the paretic arm, n (%)		
Yes	12 (18.5)	3 (8.6)
No	53 (81.5)	31 (88.6)
No response	0 (0.0)	1 (2.9)
Fitbit worn on paretic or nonparetic wrist^c^, n (%)		
Paretic	15 (23.1)	—
Nonparetic	49 (75.4)	—
Unknown	1 (1.5)	—
Fitbit worn on poststroke dominant or nondominant wrist^c^, n (%)		
Dominant	36 (55.4)	—
Nondominant	28 (43.1)	—
Unknown	1 (1.5)	—

a The 35 excluded participants include the 2 individuals that enrolled in the larger study but declined the Fitbit portion.

b Not applicable.

c Wrist placement not available for one included participant.

### Self-Reported Acceptance of Wearable Devices in Participants With Stroke

**Figure 2.**
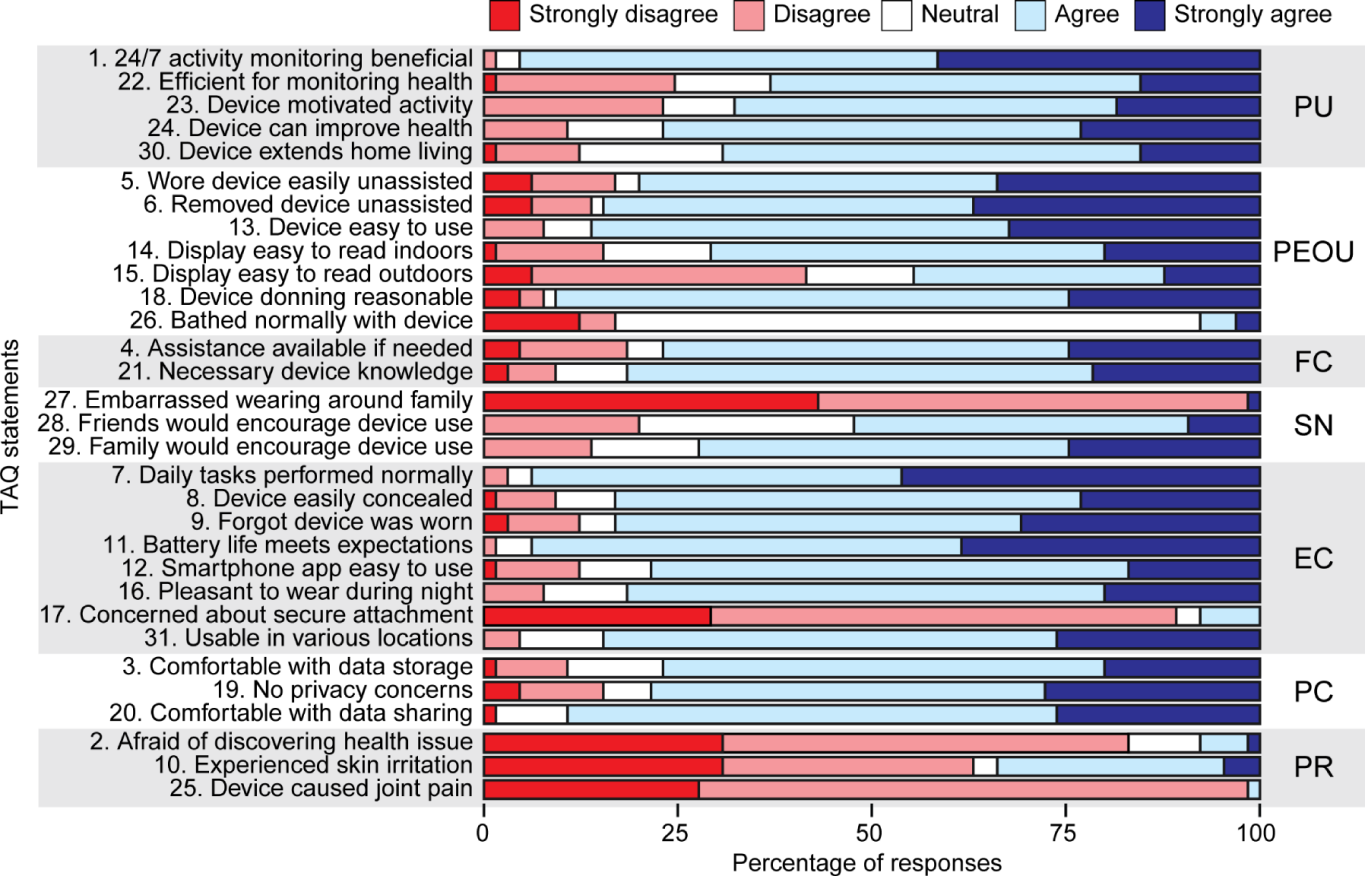
Percentage of responses to each statement on the TAQ grouped by the 7 TAQ dimensions. EC: equipment characteristics; FC: facilitating conditions; PC: privacy concerns; PEOU: perceived ease of use; PR: perceived risks; PU: perceived usefulness; SN: subjective norm; TAQ: Technology Acceptance Questionnaire.

We report percentages of responses (Strongly disagree, Disagree, Neutral, Agree, and Strongly agree) to each of the 31 TAQ statements rated on Likert scales, organized by dimension (Figure 2). The 5 statements with the highest proportions of “Agree” or “Strongly agree” responses were (in order; 2a, 2b, and so forth indicate multiple statements with the same proportions of responses): (1) Statement 1: “I think that monitoring my activity and health 24 hours a day, 7 days a week, can be a good thing;” (2a) Statement 7: “I was able to perform my daily tasks as usual while wearing the device;” (2b) Statement 11: “The battery life of the device meets my expectations;” (4) Statement 18: “I was able to put the device on in a reasonable amount of time;” and (5) Statement 20: “I am comfortable with my health data being shared with equipment manufacturers as long as it is shared anonymously.”

The five statements with the lowest proportions of “Agree” or “Strongly agree” responses were (in order): (1a) Statement 25: “Wearing the device caused me to have joint pain,” (1b) Statement 27: “I was embarrassed to wear the device in front of family members,” (3a) Statement 2: “I was afraid that the device would discover a major health issue,” (3b) Statement 17: “I was concerned that the device is not securely attached to me,” and (3c) Statement 26: “I was able to shower or bathe normally while wearing the device.”

As statements 1a-3b are reverse-coded; thus, a lower proportion of “Agree” or “Strongly agree” responses to these statements indicates a more positive perception of the device. With the exceptions of the 5 statements listed above, as well as statements 10 (“I experience skin irritations while wearing the device”) and 15 (“I find the display of the device easy to read outdoors”), a majority of participants responded either “Agree” or “Strongly agree” to the remaining 24 statements. We also note that responses to statement 26 were likely influenced by our instructions to participants to remove the device while bathing or showering.

We present group means, standard deviations (SD), medians, and interquartile ranges (IQR) of scores for each of the 7 dimensions in Table 2 (alongside the minimum and maximum possible scores for each dimension) and for each individual TAQ statement in Table 3. Medians of the summed scores for each dimension ranged from 71% in PEOU (median=25, maximum possible score=35) to 80% in all other dimensions (Table 2). Furthermore, all dimensions—with the exception of PEOU—showed median scores of 4 (“Agree”) on each individual statement (Table 3). Modestly lower scores in the PEOU dimension were largely driven by generally less agreeable responses to statement 15 (“I found the display of the device easy to read outdoors”) and statement 26 (“I was able to shower or bathe normally while wearing the device”), again noting that participants were instructed to remove the device before bathing or showering. The median for the total TAQ score was 76% of the maximum possible score (median=118, maximum possible score=155).

**Table 2.** Statistics of the responses to the Technology Acceptance Questionnaire (TAQ) dimensions and the full TAQ.

Dimension	Scores, mean (SD)	Scores, median (IQR)	Scores, possible range (min-max)
Perceived usefulness	19.1 (3.3)	20 (17-21)	5-25
Perceived ease of use	25.7 (3.9)	25 (23-28)	7-35
Facilitating conditions	7.7 (1.5)	8 (7-8)	2-10
Subjective norm^a^	11.6 (1.9)	12 (10-13)	3-15
Equipment characteristics^a^	32.5 (3.5)	32 (30-35)	8-40
Privacy concerns	11.8 (2.0)	12 (10-13)	3-15
Perceived risks^a^	11.9 (2.0)	12 (10-13)	3-15
Technology Acceptance Questionnaire	120.4 (11.8)	118 (114-128)	31-155

a These dimensions have reverse items in the subscore. Perceived risks consists of all reversed questions; therefore, the entire dimension is reversed.

**Table 3.** Statistics of the responses to each individual statement of the Technology Acceptance Questionnaire (TAQ).

Statement	Scores, mean (SD)	Scores, median (IQR)
24/7 activity monitoring beneficial	4.4 (0.6)	4 (4-5)
Afraid of discovering health issue^a^	4.0 (0.9)	4 (4-5)
Comfortable with data storage	3.8 (0.9)	4 (4-4)
Assistance available if needed	3.8 (1.1)	4 (4-4)
Wore device easily unassisted	3.9 (1.2)	4 (4-5)
Removed device unassisted	4.0 (1.1)	4 (4-5)
Daily tasks performed normally	4.4 (0.7)	4 (4-5)
Device easily concealed	4.0 (0.9)	4 (4-4)
Forgot device was worn	4.0 (1.0)	4 (4-5)
Experienced skin irritation^a^	3.6 (1.3)	4 (2-5)
Battery life meets expectations	4.3 (0.6)	4 (4-5)
Smartphone app easy to use	3.8 (0.9)	4 (4-4)
Device easy to use	4.1 (0.8)	4 (4-5)
Display easy to read indoors	3.7 (1.0)	4 (3-4)
Display easy to read outdoors	3.1 (1.2)	3 (2-4)
Pleasant to wear during night	3.9 (0.8)	4 (4-4)
Concerned about secure attachment^a^	4.1 (0.8)	4 (4-5)
Device donning reasonable	4.0 (0.9)	4 (4-4)
No privacy concerns	3.9 (1.1)	4 (4-5)
Comfortable with data sharing	4.1 (0.7)	4 (4-5)
Necessary device knowledge	3.9 (0.9)	4 (4-4)
Efficient for monitoring health	3.5 (1.1)	4 (3-4)
Device motivated activity	3.6 (1.0)	4 (3-4)
Device can improve health	3.9 (0.9)	4 (4-4)
Device caused joint pain^a^	4.2 (0.5)	4 (4-5)
Bathed normally with device	2.8 (0.8)	3 (3-3)
Embarrassed wearing around family^a^	4.4 (0.7)	4 (4-5)
Friends would encourage device use	3.4 (0.9)	4 (3-4)
Family would encourage device use	3.8 (1.0)	4 (3-4)
Device extends home living	3.7 (0.9)	4 (3-4)
Usable in various locations	4.1 (0.8)	4 (4-5)

a These statements have been reverse coded to preserve directionality.

Next, we analyzed responses to the 6 multiple-choice questions from the TAQ that did not have designated dimensions (Table 4). Most participants found the device useful, with 95.4% (62/65) rating the information provided as either “very useful” or “somewhat useful.” Nearly all participants (92.3%, 60/65) expressed willingness to continue using the device to monitor their health, and 96.9% (63/65) reported wearing the device for 15‐21 days out of the 21-day period. In terms of value, most participants indicated a willingness to pay no more than $100 for the device. Overall, 90.8% (59/65) of participants reported looking at their health data provided by their device. Finally, self-perception of activity levels varied, with 64.6% (42/65) of participants considering themselves to be active.

**Table 4.** Responses to the Technology Acceptance Questionnaire (TAQ) multiple-choice questions.

Questions and response options	Respondents, n (%)
How useful did you find the information provided by the smart wearable device (such as step count, sleep data, and heart rate) either on the wearable itself or in the smartphone application?	
Very useful	36 (55.4)
Somewhat useful	26 (40.0)
Not very useful	3 (4.6)
Not at all useful	0 (0.0)
Would you use the device you used during the last 21 days to continue to monitor or track your physical activity or health?	
Yes	60 (92.3)
No	5 (7.7)
Over the last 21 days, how often do you think you wore the smart wearable device?	
Never	0 (0.0)
Between 0 and 7 days	0 (0.0)
Between 8 and 14 days	2 (3.1)
Between 15 and 21 days	63 (96.9)
How much would you be willing to pay for the device you wore during the last 21 days?	
$0	15 (23.1)
$1-$50	17 (26.2)
$51-$100	24 (36.9)
$101-$200	9 (13.8)
$201-$300	0 (0.0)
$300-$400	0 (0.0)
Did you find yourself looking at your health data in the smartphone application more or less often after the first few days?	
No, I looked at the health data fairly consistently throughout the 21-day period	21 (32.3)
Yes, I looked at the health data more often after the first few days of use	28 (43.1)
Yes, I looked at the health data less often after the first few days of use	10 (15.4)
I did not look at my health or am not interested in monitoring it	6 (9.2)
Do you consider yourself to be an active person?	
Yes	42 (64.6)
No	23 (35.4)

### Relationships Between Self-Reported Acceptance and Fitbit Adherence

**Figure 3.**
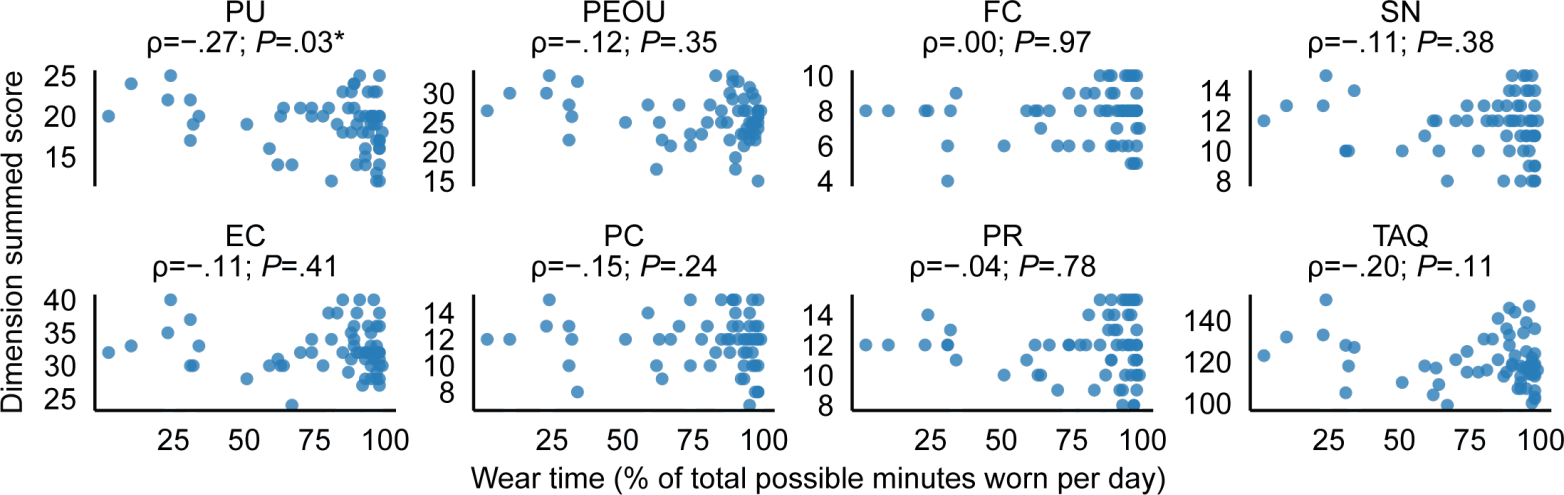
Scatterplots showing relationships between summed scores for each TAQ dimension (as well as total TAQ scores) and Fitbit wear time. Asterisks (*) indicate statistically significant relationships (*P*<.05). EC: equipment characteristics; FC: facilitating conditions; PC: privacy concerns; PEOU: perceived ease of use; PR: perceived risks; PU: perceived usefulness; SN: subjective norm; TAQ: Technology Acceptance Questionnaire.

Overall, participants wore the Fitbit for an average of 80.0% (SD 24.7%) of the total minutes in a day, with a median wear time of 91% and an IQR of 22%. Wear time exceeded the threshold needed to be considered a valid wear day on 78.0% (SD 25.8%) of days, with a median of 90% and an IQR of 33%. The scatterplots in Figures3  4 show relationships between the summed scores of the different TAQ dimensions (as well as TAQ total scores) and the percentages of wear time and valid wear days (days with ≥1080 wear minutes), respectively. Contrary to our hypothesis, there were no statistically significant positive relationships between TAQ dimension summed scores or total score and Fitbit adherence metrics. We did, however, observe 2 small but statistically significant negative associations: between PU and percent wear time (ρ=−.27; *P*=.03) and between PU and the percentage of valid wear days (ρ=−.26; *P*=.04).

**Figure 4.**
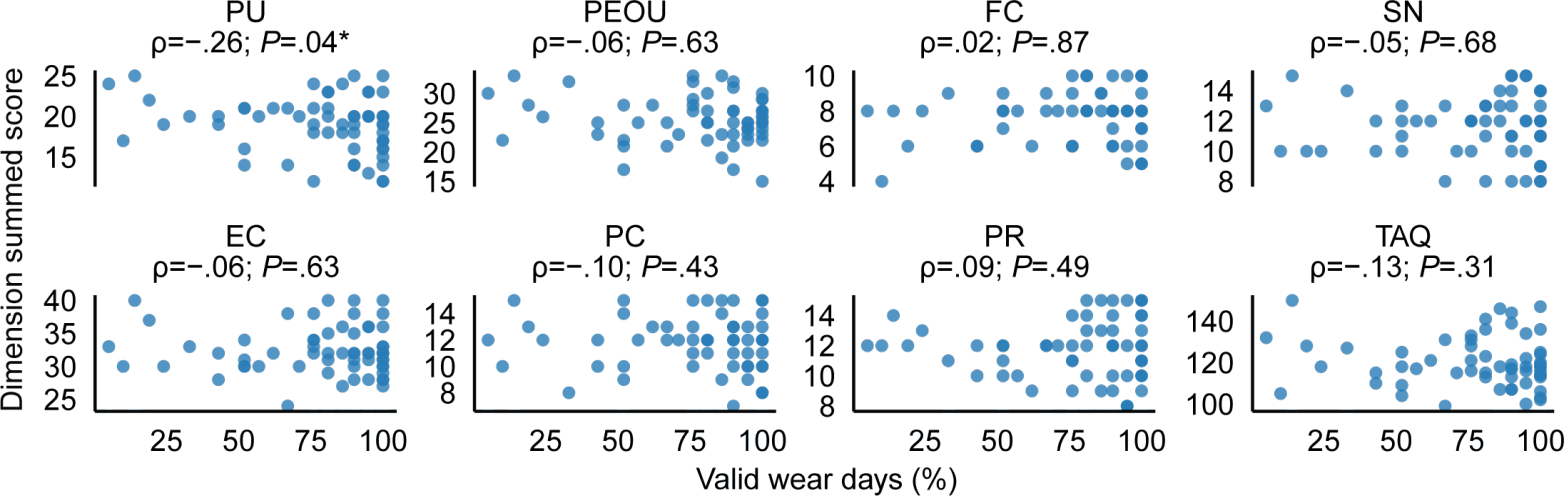
Scatterplots showing relationships between summed scores for each TAQ dimension (as well as total TAQ scores) and valid Fitbit wear days. Asterisks (*) indicate statistically significant relationships (*P*<.05). EC: equipment characteristics; FC: facilitating conditions; PC: privacy concerns; PEOU: perceived ease of use; PR: perceived risks; PU: perceived usefulness; SN: subjective norm; TAQ: Technology Acceptance Questionnaire.

### Open-Ended Comments on the Devices

Participants provided a series of positive and negative comments about their experiences with the devices. Themes of positive comments included the ability to monitor real-time heart rate, the use of step count data as motivation to increase activity, and the provision of sleep data. Themes of negative comments included difficulties donning and doffing the device, technical challenges with the associated mobile app, difficulty reading the screen on the device, and concerns that other wearables and smartwatches might provide more relevant or more comprehensive data.

Qualitatively, we did not find any consistent themes in the open-ended comments that related to adherence to wearing the device. For example, among participants who wore the device for fewer than 50% of possible minutes (n=8), we observed that most of the comments were largely positive in nature. These participants reported that the Fitbit provided useful step count information, helped them stay on track with physical activity, and offered useful sleep data. The only negative comments were centered around the potential utility of additional training for using the device and the small size of the device screen.

## Discussion

### Principal Findings

Our study examined self-reported perceptions of the Fitbit Inspire 2 wearable activity monitor among individuals with stroke, as measured by the TAQ. A majority of the participants thought the device was beneficial for their health, efficient for monitoring their health, easy to use and to don and doff, and unintrusive to daily life; one notable exception was the response to the statement “I find the display of the device easy to read outdoors.” Generally, participants did not express significant concerns about privacy or data security, consistent with previous studies [30,31]. Contrary to our hypothesis, more agreeable responses to the TAQ statements were not associated with higher average daily wear time and valid wear days at a statistically significant level.

### Comparison With Previous Work

The findings of our study are consistent with previous literature demonstrating acceptance of wearable activity monitors in other populations [13,32-36]. Given the growing interest in using wearables for activity monitoring and telerehabilitation after stroke [26,37-40], it is important to consider not only their potential benefits but also the potential challenges related to low levels of physical activity and cognitive and motor impairments that may affect this population’s acceptance and engagement [10,41,42]. There were no commonly reported acceptance concerns that we deemed likely to be related to poststroke motor impairment. For example, participants widely agreed with the statements “I was able to wear the device easily without help from another person,” “I find the device easy to use,” “I was able to put the device on in a reasonable amount of time,” and “I was able to remove the device easily without help from another person.” These findings complement recent studies demonstrating the perceived value and user satisfaction of wearable technologies in stroke rehabilitation [43-45] and support a path toward scalable implementation of remote monitoring with wearable devices. This is likely due in part to the flexibility we allowed in permitting participants to wear the device on their paretic side if needed, accommodating individual motor abilities.

We also highlight that the study participants reported generally agreeable responses across all 7 dimensions of the TAQ. Previous work highlighted that technical and usability issues (eg, requiring a mobile app to sync the data from the device to the server) may affect the PU of wearable devices [16]; however, we did not observe this in our sample. This is potentially because any technical difficulties were often addressed via interactions with the study team. Our findings across the different dimensions were largely similar to those reported in a previous sample of older adults [13]. It is important to emphasize that monitoring of device adherence may be necessary despite the high reported acceptance. Our findings did not support the hypothesis that higher user acceptance as measured by the TAQ would correlate with adherence to wearing the device, as we did not observe statistically significant positive correlations between TAQ responses and our measures of adherence. Furthermore, we found that 5 participants reported that they had worn the device for at least 15 of the preceding 21 days (in response to the multiple-choice question) but provided fewer than 15 days of Fitbit data. This revealed that high reported acceptance of the device does not guarantee that a patient or research participant will necessarily adhere to wearing the device in everyday life. Technologies that help to automate oversight of device wear and messaging to promote adherence will be important for ensuring data quality [46].

The correlational analyses indicated small but significant negative associations between the PU score and both adherence metrics. These results are contrary to our hypotheses, which were grounded in the technology acceptance model and related literature, where PU is typically positively associated with adoption behaviors [47,48]. Existing studies have shown that higher PU is often associated with sustained use of technology across various domains [49-51], including health care settings [52-55]. Our findings may be attributed to the specific context in which the wearable device was used. Unlike most previous technology adoption studies where participants voluntarily adopted technology based on its usefulness, our study cohort used the Fitbit as part of their participation in a research study. This mandated context could have influenced PU differently from typical motivational factors driving technology adoption, as individuals may have rationalized their behaviors based on the rewards of participating or the consequences of noncompliance. It is possible that participants did not view the Fitbit as inherently useful for their health recovery goals but instead perceived it as a tool for fulfilling study requirements. They may have also overreported PU due to social desirability (ie, aiming to please the study team). While survey responses suggest that most participants agreed that the device could improve health and monitor well-being efficiently, these endorsements may reflect a general perception of health technology utility rather than a personalized alignment with stroke recovery needs. Consequently, their assessment of PU may reflect this externally driven motivation rather than genuine alignment with personal health management goals.

As the push toward clinical use of wearables in stroke rehabilitation continues to move forward, it is also important to consider the needs and perspectives of all key stakeholders—patients, their family members and caregivers, and clinicians—in addition to the device acceptance reported by participants in our study. Recent studies have provided vital information regarding how persons with stroke prefer to receive data from wearables and identified a set of metrics deemed most useful [23]; incorporated perspectives from patients and clinicians on the value of using wearables and identified preferences for incorporation into clinical care [44]; and demonstrated important design considerations for adoption of the wearables and accompanying smartphone apps as outlined by persons with stroke [45]. For example, considering the difficulty of donning and doffing, an alternative strap such as a Velcro strap instead of the original buckle band may improve usability. Future work should consider these multifaceted aspects—patient acceptance, patient and clinician data provision preferences, and device design—as we move closer to clinical implementation of wearables in stroke rehabilitation.

### Limitations

We acknowledge some limitations in our study. First, our sample was heterogeneous regarding stroke chronicity. Accordingly, we did not design this study to assess how wearable device acceptance may differ across stages of stroke recovery (eg, acute, subacute, and chronic). However, this diversity in stroke recovery stages could be beneficial, as it reflects the clinical reality in which wearable devices in stroke rehabilitation should not discriminate based on recovery stage but rather be accessible and valuable to patients at various points in their recovery. Second, we only used the Fitbit Inspire 2 device. While we anticipate that many of our findings may generalize across different models of commercially available wearable devices due to the nature of the statements included in the TAQ, we do not have data to support this directly. Third, we focused on the TAQ to provide information about device acceptance in particular. We did not use other instruments that could provide additional data on other aspects of patient perceptions about wearable devices (eg, the System Usability Scale for assessment of usability). Finally, our study focuses on individuals who own smartphones and have home Wi-Fi. This digital access criterion may have biased the sample toward more tech-savvy or higher-functioning individuals.

### Conclusion

This study reported the perceived acceptance of a wrist-worn activity monitor among persons with stroke. In response to statements on the TAQ, participants with stroke generally reported the device to be beneficial for their health, useful for monitoring their health, easy to use, and minimally intrusive. We observed generally agreeable responses to TAQ statements across all 7 dimensions of the instrument. Contrary to our hypothesis, more agreeable responses to the TAQ statements were not positively correlated with metrics of device wear, indicating that adherence to wearing the device should not be assumed even when participants report high device acceptance. In summary, this study provides new information about the acceptance of wearable activity monitors among persons with stroke and its association with real-world device wear.

## Abbreviations

ICD-10: International Classification of Diseases, Tenth Revision
PEOU: Perceived ease of use
REDCap: Research Electronic Data Capture
TAQ: Technology Acceptance Questionnaire
